# Virtual Vanderbilt Summer Science Academy highlighted the opportunity to impact early STEMM students career knowledge through narrative

**DOI:** 10.1371/journal.pone.0258660

**Published:** 2021-11-10

**Authors:** Kendra H. Oliver, Christina Keeton, Roger Chalkley, Elizabeth Bowman

**Affiliations:** 1 Department of Pharmacology, Basic Sciences, School of Medicine, Vanderbilt University, Nashville, TN, United States of America; 2 Communication of Science and Technology Program, College of Arts and Science, Vanderbilt University, Nashville, TN, United States of America; 3 Initiative for Maximizing Student Diversity, The Office of Biomedical Research and Education, Basic Sciences, School of Medicine, Vanderbilt University, Nashville, TN, United States of America; 4 Vanderbilt Summer Science Academy, The Office of Biomedical Research and Education, Basic Sciences, School of Medicine, Vanderbilt University, Nashville, TN, United States of America; 5 Senior Associate Dean, The Office of Biomedical Research and Education, Basic Sciences, School of Medicine, Vanderbilt University, Nashville, TN, United States of America; 6 Graduate Programs in Biomedical Sciences, The Office of Biomedical Research and Education, Basic Sciences, School of Medicine, Vanderbilt University, Nashville, TN, United States of America; Universidade da Coruna, SPAIN

## Abstract

Due to COVID-19 precautions, the Vanderbilt University summer biomedical undergraduate research program, the Vanderbilt Summer Science Academy (VSSA), rapidly transitioned from offering an in-person training program to a virtual seminar format. Our program typically supports undergraduate development through research and/or clinical experience, meeting with individuals pursuing postgraduate training, and providing career development advice. Evidence supports the idea that summer programs transform undergraduates by clarifying their interest in research and encouraging those who haven’t previously considered graduate studies. We were interested in exploring whether a virtual, synchronous program would increase participants’ scientific identity and clarify postgraduate career planning. Rather than create a virtual research exposure, our 5-week "Virtual VSSA" program aimed to simulate the casual connections that would naturally be made with post-undergraduate trainees during a traditional summer program. In seminars, presenters discussed 1) their academic journey, explaining their motivations, goals, and reasons for pursuing a career in science as well as 2) a professional story that illustrated their training. Seminars included Vanderbilt University and Medical School faculty, M.D., MD/Ph.D., as well as Ph.D. students from diverse scientific and personal backgrounds. In addition, weekly informational sessions provided an overview of the nature of each degree program along with admissions advice. Through pre-and post-program surveys, we found that students who registered for this experience already strongly identified with the STEMM community (Science, Technology, Engineering, Mathematics, and Medicine). However, participation in the Virtual VSSA increased their sense of belonging. We also uncovered a gap in participants’ understanding of postgraduate pathways prior to participation and found that our program significantly increased their self-reported understanding of postgraduate programs. It also increased their understanding of why someone would pursue a Ph.D. or Ph.D./MD versus M.D. These changes did not uniformly impact participants’ planned career paths. Overall, by providing personal, tangible stories of M.D., MD/Ph.D., and Ph.D. training, the Virtual VSSA program offered seminars that positively impacted students’ sense of belonging with and connection to the STEMM disciplines.

## Introduction

Undergraduate summer research programs are an opportunity for burgeoning scientists to experience research full-time, often at research-intensive institutions. Evidence supports the idea that summer programs transform undergraduates by clarifying their interest in research and encouraging those who haven’t previously considered graduate studies [1]. The benefits of in-person research experiences have been investigated through both qualitative and quantitative research [2, 3]. Overall, undergraduate research opportunities increase understanding, confidence, awareness, as well as clarify interest in STEMM careers and in pursuing a Ph.D. degree [2–6]. Additionally, it is clear that the longer duration and variety of research experiences are related to positive outcomes [5]. Summer undergraduate research experiences impact students’ retention and career advancement in science. It is also clear from other studies that having a strong science identity is one of the only good predictors of moving into a science-related career field after graduation [7]. Additionally, previous literature suggests that scientific identity and belonging can be built through non-research interactions. Science identity is defined as the student’s alignment of their perception of a career with one or more of their personal identities. If they cannot see someone like them doing a job, it is less likely that they will follow that particular path [8]. Therefore, providing opportunities that expand students’ images of the roles available in science is key [9, 10]. Additionally, mentoring and mentorship plays a significant role in developing science identity [11, 12]. Through developing mentor-mentee relationships, the students can integrate their research experiences with the beliefs and expectations to excel in scientific careers.

In the face of the global COVID-19 pandemic, many laboratories were closed or operating at restricted capacity in 2020 and thus most undergraduate students were deprived of an in-person summer research experience. For these current undergraduate students, unable to attend in-person research experiences, we felt that it was critical to continue to provide opportunities to interact with the science community and potentially foster a more profound interest in science. Is it known that representation plays a role in students sense of belonging [13]. This is true for a number of environmental factors including a socioeconomic status where participating rely on access to cultural, social and science capitals [14]. Previous studies have demonstrated how moderate gaps in science achievement related to both household income and parental education [15]. Beyond opportunity, students lower in social status may also experience rejection sensitivity in evaluative assessment situations that emphasis rank and status, so increasing opportunity can help beyond simply providing experience [16]. In order to increase the representation of underrepresented and disadvantaged students, exposure to the STEMM community and a diverse array of mentoring experiences are necessarily [17]. The goal of the program was to break preconceived notions that individuals who pursue postgraduate training are highly homogeneous and instead demonstrate the diversity of the community in order to increase participant’s own sense of identity and belonging to STEMM.

The Vanderbilt Summer Science Academy (VSSA) is a summer research program that hosts approximately 100 students each summer from institutions across the country. During a typical year, the VSSA offers weekly research seminars, a wide range of enrichment activities and lectures, as well as social activities for those undergraduates engaged in research on our campus during the summer. The COVID-19 pandemic in 2020 triggered a mandatory closure of Vanderbilt’s in-person summer programming for undergraduates. In response, we set out to design a virtual summer experience that could increase students’ sense of identity and belonging in science. Rather than simulating laboratory research, our goal was to create a virtual seminar series that focused explicitly on telling personal narratives of those in the sciences and as well as discussing post-graduate degree program expectations.

We hypothesized that this seminar series would increase students’ sense of belonging. Previous literature supports the notion that scientific identity can be built through non-research interactions [7]. However, no study has specifically analyzed the effectiveness of virtual seminars in supporting scientific identity. To assess whether our virtual program accomplished these goals, we analysed pre- and post-survey responses from students who participated in the virtual VSSA program to asses scientific identity, understanding of degree opportunities, and interest in these paths. We also examined how a participant’s sense of identifying with the speaker could impact their interest in a particular postgraduate degree pathway.

## Materials and methods

This study and the data used in this study were approved by the Vanderbilt behavioral and social sciences IRB review committee (approval #201965). The data were analyzed anonymously.

### Program design and recruitment

The design of the program is shown in S1 Fig. This program was intentionally designed to expose students to M.D., MD/Ph.D., and Ph.D. career paths through linking personal narratives, research stories, and program admissions exposure. Program administrators recruited faculty from diverse backgrounds to be presenters. Graduate and medical students in all three tracks were also recruited based on their diverse backgrounds. The student participant recruitment method is also shown in S1 Fig. Using the VSSA applicant pool, 1167 students were emailed about the program. Of those emailed, 517 registered for the virtual VSSA program. Students who registered for the program were sent a separate email inviting them to complete the pre-survey and this same group was emailed after the program to complete the post-survey. The pre-survey was completed by 346 students and 136 completed the post-survey. We had an average of 101.27 participants attend each session (range 66–220). There were no significant differences in demographic or background experience between participants who completed both surveys or those that completed just the entry survey (S1 Table, “Just Pre Survey” vs “Pre- and Post- Survey).

Data were collected immediately after each presentation: In order to correlate identity in a speaker with interest in a specific career path, we administered a simple 2-question survey at the end of each seminar, presented on Zoom. These questions were “How much do you identify with this speaker”, with responses “Strongly identify”, “Somewhat identify”, “Neutral”, “Minimally identify”, “Did not identify” and “How interested does this seminar make you in this path?” with responses “Very interested”, “Somewhat interested”, “Neutral”, and “Not at all interested”. We related the bulk responses to these questions for all participants across all seminars (n = 668) (Table 2).

### Survey

**Fig 1:**
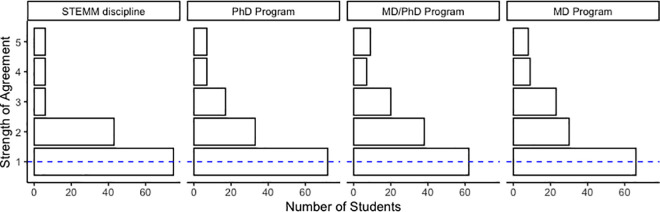
The Virtual VSSA program effectively provides participants exposure to the STEMM community. In the post-survey, participants were asked to what extent they agreed with the statement, "I learned a lot about xxx through the Virtual VSSA seminar series.” This question was posed about (A) STEM disciplines, (B) Ph.D., (C) M.D./Ph.D., and (D) M.D. programs. Students responded on a Likert scale indicating their level of agreement. The number of responses for each category is shown. The median response is indicated with the blue line.

We utilized a modified version of the Scientific Identity Scale [18], which included 5-items on a Likert scale. Participants were asked to assess on a scale of 5 (strongly disagree) to 1 (strongly agree) to what extent each statement was true of them. Items included "I have a strong sense of belonging to the community of scientists," "I derive great personal satisfaction from working on a team that is doing important research," "I have come to think of myself as a ’scientist,‴ "I feel like I belong in the field of science," and "The daily work of a scientist is appealing to me." The scale had high internal consistency (α = .86). The survey instrument used for this study can be reviewed in S2 Table. Note that in our scale (1 = strongly agree, 2 = agree, 3 = neutral, 4 = disagree, 5 = strongly disagree), a lower value means stronger agreement with the given statement. In the post-survey, we also asked participants to rate their learning of these areas (Fig 1).

### IRB approval

This study and the data used in this study were approved by the Vanderbilt behavioral and social sciences IRB review committee (approval #201965).

### Data analysis

Data were analyzed using R Studio (RStudio Team (2020). RStudio: Integrated Development for R. RStudio, PBC, Boston, MA URL http://www.rstudio.com/.) and the tidyverse, ggplot2, and reshape2 packages. Likert scale data were treated as ranked categorical data. The paired data (data of pre- and post-surveys completed by the same student) were analyzed using a Wilcoxon ranked sum test. To compare differences in responses between demographic groups, responses were divided into two groups: group 1 consisted of only individuals who recorded a “positive change”, and group 2 was made up of all other responses. Chi-Squared test of independence analyses were performed to identify potential relationships between these groups.

## Results

### Overall impacts of program

We were interested in determining whether students who participated in this virtual program developed an increased sense of belonging in the science community and in an understanding of STEMM careers by first analyzing the post-survey responses only. Overall, students reported in the post-survey a strong level of agreement that they learned more about STEMM (Fig 1). Despite this being a short, 5-week program, the majority of students “strongly agree’’ that they learned about the STEMM discipline, and the STEMM Ph.D., MD/Ph.D., and M.D. programs.

In order to dissect this further and get a deeper sense of how participation in the program impacted their scientific identity, program knowledge, and career interest, we compared individual students paired pre-and post-program survey data. Table 1 shows the p-value for change in individuals pre- versus post-surveys response using the Wilcoxon test. Based on the results of the surveys, the program increased a sense of belonging in the STEM community, postgraduate degree knowledge, and interest in M.D./Ph.D. degree paths (Table 1; S2 Fig). Specifically, students had an increased positive response to the statement, "I have a strong sense of belonging to the STEMM community." Within the postgraduate degree knowledge category, all survey questions had a statistically significant increase in positive agreement. Finally, in the career intentions category, there was a statistically significant increase in the number of students who positively responded to the question "How strongly are you considering pursuing an M.D./Ph.D.?" but there was no change in students’ responses to the prompt that asked about the students interest to pursue Ph.D. or M.D. programs.

**Table 1:** Overall, the program increased a sense of belonging in the STEMM community, postgraduate degree knowledge, and interest in M.D./Ph.D. degree paths.

Survey Question	Wilcoxon p-value
**Science Identity**	
I have a strong sense of belonging to the STEMM community	0.009
I have come to think of myself as a ‘scientist’	0.058
I feel like I belong in the STEMM field	0.256
I derive great personal satisfaction from working on a team that is doing important research	0.302
The daily work of a scientist is appealing to me	0.405
The daily work of a physician is appealing to me	0.732
**Post-graduate Degree Knowledge**	
I have an understanding of the program structure for a PhD program	2.27E-12
I have an understanding of the program structure for an MD/PhD program	2.80E-11
I have an understanding of the program structure for an MD program	6.29E-07
I have an understanding of career outcomes for individuals with a PhD	1.26E-09
I have an understanding of career outcomes for individuals with an MD/PhD	1.16E-09
I have an understanding of career outcomes for individuals with an MD	0.002
I understand why individuals would want to pursue a specific post-graduate science degree (PhD vs MD/PhD vs MD)	6.30E-07
**Career Intentions**	
How strongly are you considering pursuing a PhD?	0.259
How strongly are you considering pursuing an MD/PhD?	0.011
How strongly are you considering pursuing an MD?	0.800

**Fig 2:**
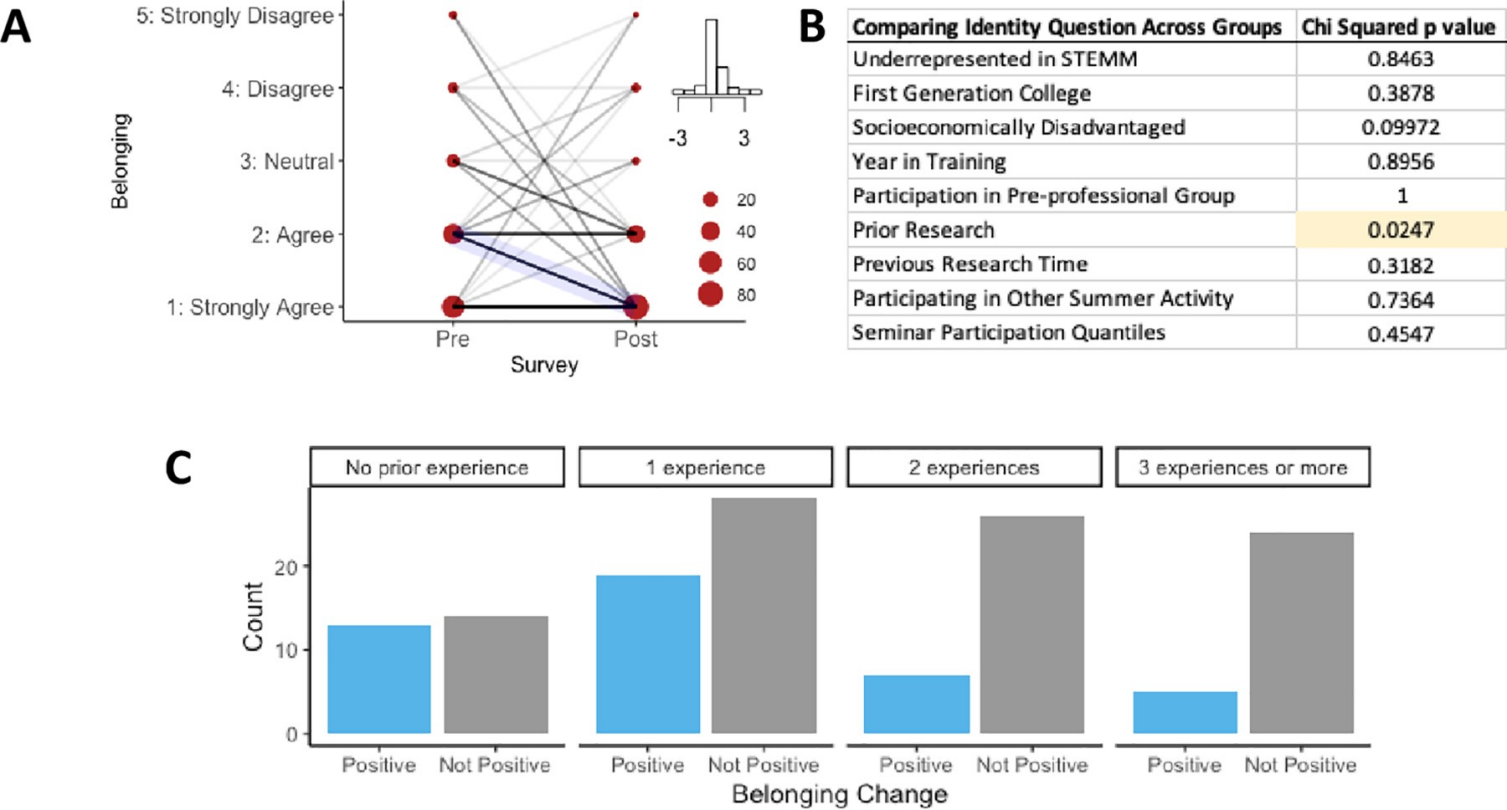
While most students started the program with a strong sense of belonging, students with a positive change in the sense of belonging had less prior research experience. (A) We examined student’s responses to the prompt, "I have a strong sense of belonging with the STEMM community." Responses are shown based on a 5-point Likert scale, with one indicating that they strongly agreed with the statement to five indicating that they strongly disagreed with the statement. The inset is a histogram of the change in values (pre-test minus the post-test value) with the highest bar at 0, indicating no change in response. (B) Demographic information in individuals with positive (improved) “Belonging” change compared with those without a positive change were analyzed using a Chi-squared analysis. (C) The positive (improved) change versus non-positive change in belonging were compared with amount of research experience, no prior experience, one, two, or three or more experiences.

**Fig 3:**
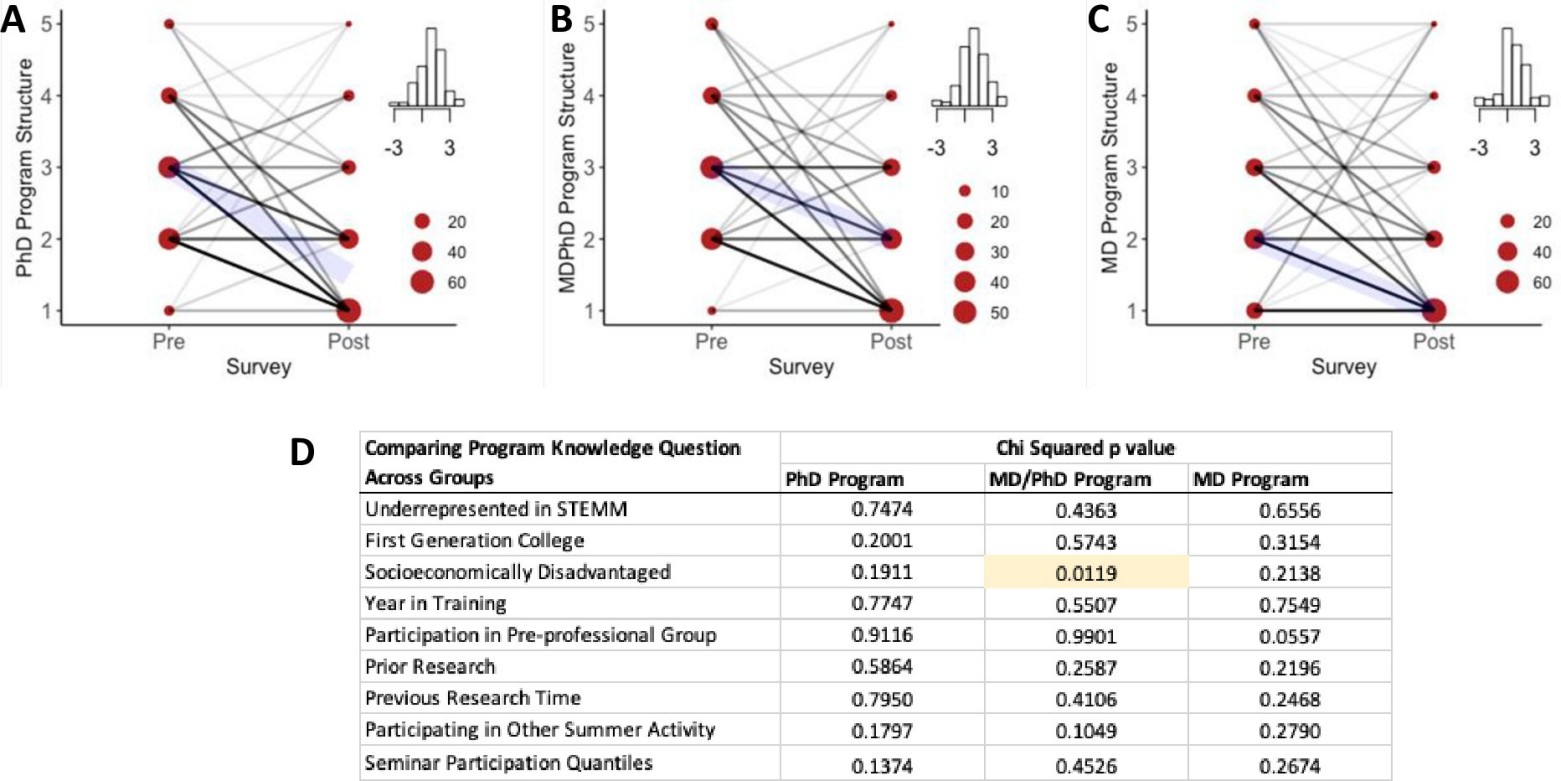
Participation in the virtual VSSA program increases Ph.D., MD, MD/Ph.D. program knowledge. (A-C) The inset is a histogram of the change in values (pre-test minus the post-test value) with the highest bar at 0, indicating no change in response. Positive changes indicate increased agreement with the statement. (A) First, we examined student responses to the prompt "I have an understanding of the program structure for a Ph.D. program." The average pre-survey response was "neutral," but following the program, most students responded between "agree" to "strongly agree." (see purple highlight line, indicating median value for each survey). (B) Similarly to the prompt "I have an understanding of the program structure for an M.D./PhD program," student pre-survey response was "neutral" however, following the program, most students responded "agree." (C) To the prompt, "I have an understanding of the program structure for an MD program," in the pre-survey, most students responded "agree," but following the program, most students responded, "strongly agree." (D) Finally, we examined only the groups who had a positive change in understanding of the Ph.D., MD/Ph.D., and M.D. programs with positive identification with the groups listed on the first column. There was a positive correlation between a positive change in belonging score with socioeconomically disadvantaged students (P = 0.012).

Comparing pre-and post-surveys, we examined how students’ responses to the survey questions changed. Students responded to these prompts using a 5-point Likert scale, with one indicating that they strongly agreed with the statement and five indicating that they strongly disagreed with the statement. Comparisons of the pre- versus post-survey comparisons were made using a Wilcoxon test. Significant differences in the pre-and post-survey responses, highlighted in yellow. See Fig 2, S2 Fig, Fig 3, and S4 Fig for individual response changes.

### Belonging in STEMM

We were interested to learn more about students’ sense of belonging and how this related to participation in the Virtual VSSA program. Most of our participants already started the program with a strong sense of belonging: Almost half of the participants rated their belonging very high as indicated by "agree" or "strongly agree" to the prompt "I have a strong sense of belonging to the STEMM community." (Fig 2A, Pre-survey). However, following participation in the program, individuals demonstrated a modest yet significant increase in their sense of belonging in STEMM (Fig 2A, decrease in the Likert scale; note inset histogram of changes; p = 0.009, Table 1). We next aimed to determine if individuals who had increases in belonging had different demographic or experiential backgrounds from those that did not. In order to understand the statistically significant difference observed in the summary data shown in Table 1, we stratified the pre-/post-survey belonging data into positive (improved) and not positive change groups. Using a Chi-squared analysis, we examined whether there was a positive change associated with a number of factors including underrepresented status in STEMM, first-generation college students, socioeconomic disadvantage, years in college, participation in a pre-professional group, prior research, previous research time (number of semesters), participating in other summer activities and seminar participation qualities (Fig 2B). We observed that the only component of participants’ background that significantly influenced an increased belonging was their previous research experience, specifically students with limited research reported the greatest gain in belonging. Those with less prior research experience were more likely to see a positive change in the sense of belonging as compared to students who had previous research experience (Fig 2C). In particular, the highest increases in the sense of belonging were seen in students that have limited experience in research (Fig 2C). All other demographic categories were not associated with different changes in STEMM belonging (S3 Fig).

### The intersection of identity and interest

The Virtual VSSA program provided participants with diverse, tangible examples of individuals on postgraduate paths so that they could better determine whether they belong in the field. To explore the success of this program, we examined how a participant’s sense of identifying with the speaker could impact their interest in a particular postgraduate degree pathway. Our hypothesis was that if a participant identified with a speaker more strongly, they would be more interested in a given path. Immediately following each seminar, students were asked, "How much do you identify with this speaker?" and "How interested does this seminar make you in this path?". These data were aggregated and analyzed based on the correlation between identification with speakers and interest in the career pathway. We were surprised that in this bulk analysis, so many students identified strongly with speakers. This also was associated with interest in the career pathway of the speaker (Table 2). This strong sense of identity and interest in paths led us to further explore the participants’ understanding of postgraduate paths.

**Table 2:** There is a positive association between identifying with the speakers and interest in a career pathway.

		How much do you identify with this speaker?				
		Did not identify	Minimally identify	Neutral	Somewhat identify	Strongly identify
How interested does this seminar make you in this path?	Not at all interested	0.15	0.60	0.75	0.75	0.15
	Neutral	0.90	2.85	5.10	3.90	0.60
	Somewhat interested	0.00	1.05	5.70	20.09	5.55
	Very interested	0.15	0.90	3.60	20.54	26.69

Association between participants’ level of interest in, and identification with the speakers of, online seminars in an undergraduate summer research program. After each speaker in the seminar series, we asked, “How much do you identify with this speaker?” and “How interested does this seminar make you in this path?". We explored the correlation of these two questions with the percentages that answered each question indicated. Color coding was used to show the extent of the total percentage that fell within each group, with the darker yellow indicating an increased percentage of responses.

### Understanding of Ph.D., MD/Ph.D., and M.D. programs

One of the most striking effects of the program was the gain in Ph.D., MD, MD/Ph.D. program knowledge for participants. First, we were surprised to uncover a lack of participants’ understanding of post-graduate paths (Fig 3A–3C, Pre-survey). This was interesting because these participants felt a strong identity with STEMM at this same time, before participating in the program (Fig 2A). However, through the Virtual VSSA, participants in the program showed significant positive gains in Ph.D. (Table 1; Fig 3A), M.D. (Fig 3B), and M.D./Ph.D. (Fig 3C) program knowledge. These gains were in the absence of an on-site summer research program, and there were no significant differences if students had previously performed research (Fig 3D). In fact, the only demographic or background category that resulted in a difference in these program knowledge changes was the impact of a participants’ socioeconomic background on their understanding of M.D./Ph.D. programs (Fig 3D). However, in looking at the change in median score, this appears to have a minor impact (S4H Fig). Participants also had an increased understanding of careers following these post-degree paths (S4A–S4C Fig) and motivation for why anyone pursues each path (S4D Fig). While this program did seem to change some participant’s interest in post-graduate paths, these changes were not uniform across participants and likely reflect an individualized response to understanding these programs in more detail (S4E–S4G Fig). We consider this another success of the program.

**Fig 4:**
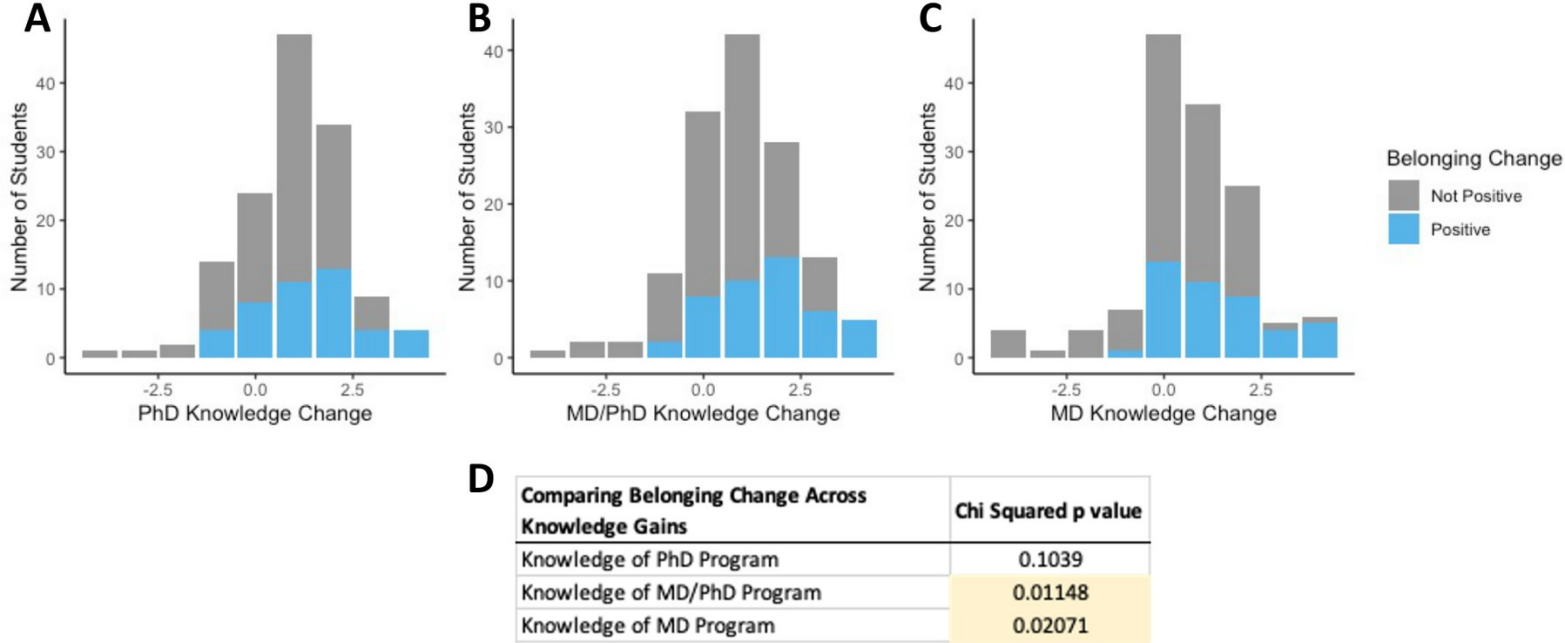
Students who had a positive change in STEMM community belonging had a larger increase in Ph.D., MD, and M.D./PhD program knowledge. (A) Positive STEMM belonging changes have to increase Ph.D. program knowledge, (B) increase M.D./PhD program knowledge, (C) and increase M.D. program knowledge. (D) We compared belonging changes across program knowledge gains and found a positive association between students’ sense of belonging and M.D./PhD and M.D. programs using a chi-squared test of independence.

Given that we observed increases in *both* participants’ sense of belonging to STEMM *and* their understanding of post-degree paths, we investigated whether these categories were related using a chi-square test of independence. Specifically, we stratified the individuals with a positive change in STEMM belonging from those that did not have a positive change and mapped this on their change in program knowledge. Strikingly, there was an increased ratio of students that also reported an increased sense of belonging within the STEMM community (Fig 4A–4C, a shift in blue towards the right). In other words, students who had a positive change in STEMM community belonging had a more considerable increase in Ph.D., MD, and M.D./Ph.D. program knowledge. Statistically, this was particularly true for M.D./Ph.D. (p = 0.011) and M.D. (p = 0.021) programs; however, the Ph.D. knowledge (p = 0.104) trended this way as well (Fig 4D).

## Discussion

Through personal experience leading the summer program in the past, and our own experiences, we find that personal stories make being a scientist relatable. With the restrictions cause by the COVID-19 pandemic, we had the unique opportunity to untangle the impact of programming and identity from doing bench research. One aspect of scientific identity is the students’ sense of belonging within the scientific community. Importantly, we uncovered a substantial gap in undergraduate students’ understanding of postgraduate program structure. Fortunately, a virtual program like the Virtual VSSA provides a straightforward opportunity for institutions that offer postgraduate programs to address this gap in understanding.

Our program data suggest a positive impact on students’ sense of belonging to the STEMM community, a small but significant aspect to developing their scientific identity. A rich body of research highlights how fostering a sense of belonging is key to allowing a diverse undergraduate population of STEMM majors to flourish. Research has demonstrated that cultural dissonance, not a lack of ability or interest, is what prompts students to leave undergraduate programs in STEMM [19]. We were hoping that the structure of our program would allow participants to hear researchers’ personal narratives and relate to these journeys. With an increased sense of connectivity and commonality with the research, we hypothesized we would observe increased identity with the STEMM discipline and community. This suggests that even through virtual platforms, students’ sense of belonging can be increased through the process of personal narrative storytelling.

More specifically, our data identifies that virtual personal narrative mixed with scientific content talks may be particularly impactful for students who have not had previous research experience. This is likely a reflection that individuals who have not performed much research started the program with a weaker sense of belonging; however, even a virtual seminar series can be impactful. It is clear that students experience different gains and benefits at different stages of their undergraduate research experience. Previous data would suggest that longer experiences offer students more benefit [20]. However, programs that help reduce concerns about belonging have been particularly effective at reducing achievement gaps [21, 22]. For example, the Walton and Cohen study aimed to lessen psychological perceptions of threat on campus by framing social adversity as common and transient, leading to adversity as an indictment of their belonging [22]. In another study, showcasing how diverse backgrounds shape the college experience, as compared to similar stories without mentioning the person’s background, eliminated the social-class achievement gap by increasing first-generation students’ tendency to seek out college resources, and their end-of-year grade improved as a result [23]. As described above, many participants already started the virtual VSSA program with a strong sense of STEMM belonging. However, the sense of belonging increased significantly for those with less than one year of research experience. Interestingly, the undergraduate year did not correlate with belonging increases, highlighting the impact of a program like this across all training levels. Moving forward, we would particularly recommend personal narrative storytelling focused on diversity to encourage social belonging for students who are in the early training phases in STEMM disciplines.

Clearly, the program was useful in educating students about the expectations and pathways for postgraduate careers. This came as no surprise as many have noted the barriers and lack of support structures as students navigate through the application phase, particularly Latino [24] and first-generation college students. English and Umbach defined a model for student graduate school applications that comprises three phases; (1) an individual develops an aspiration for graduate-level education, (2) the student submits applications to graduate schools, and (3) the student enrolls in a graduate program [25]. Our programming suggests that students highly valued information about the application process. This is supported by evidence that participation in undergraduate research programs and support from institutional program managers can help students successfully navigate the graduate school application process [24]. However, we also found that the programming increased participants’ interest in pursuing an M.D./Ph.D. career pathway but did not significantly change their consideration for Ph.D. or M.D. programs. Because program knowledge gains were so strong, it would appear that more overall programming is needed to share personal narratives and a sense of community within scientific training.

Our data also illustrate that students who had a positive change in STEMM community belonging had a larger increase in Ph.D., MD, and M.D./Ph.D. program knowledge. It is likely that these two variables are related. As a student gains knowledge about the process of applying and pursuing postgraduate STEMM training, they are more likely to feel a sense of belonging within the community. Overall, these data would suggest that exposure and discussion around the post-graduate program knowledge can also increase the student’s sense of belonging in STEMM and future educational pursuits.

Overall, the results of this study demonstrates how virtual summer programs can benefit students who cannot do summer research programs or are in unable to be in a in-person setting. Even when in-person programming commences, this type of program design may be used to enhance a sense of belonging for students, particularly those early in their STEMM careers. Next steps could include comparing the degree of belonging from seminars as compared to the casual relationships formed during in-person experiences. However our data clearly demonstrate that virtual meetings, on their own, can provide an added sense of scientific identity and community. The benefits gained by participants were no different between students who had other summer experiences, showing that this programming could be beneficial even when normal summer research commences. We highly recommend that R1 research institutions provide this type of experience and partner with non-R1 colleges and universities as a way to expand inclusivity and belonging in STEMM through the virtual, low-cost, non-capacity restrictive medium. Recordings of our series are available for any student or program to incorporate in their curriculum, and we aim to host a similar series for the 2021 season (https://medschool.vanderbilt.edu/vssa/virtual-vssa-2020/).

## Supporting information

S1 Fig Program overview. (A) To begin advertising the virtual VSSA program, the 1167 VSSA applicants were invited to participate. Of this group, 517 registered for the virtual VSSA sessions. Three hundred forty students completed the pre-survey while 136 completed both the pre-and the post-survey. This was the final population analyzed in this study, which represented just over 10% of the entire pool of VSSA students initially contacted. (B) Here we show a list of program elements by week. The program was a five-week program that began June 1^st^. There were five components: Faculty seminars, MSTP student seminars, structure and applications, Ph.D. student seminars, and M.D. student seminars. There was also a final presentation on resume and CV building that was delivered asynchronously. All other meetings were held synchronously.
(TIF)

S2 Fig Participant identity. We examined student’s responses to the prompts regarding their scientific identity (See S2 Table for specific questions). Responses are shown based on a Likert scale, with 1 indicating that they strongly agreed with the statement to 5 indicating that they strongly disagreed with the statement. Purple highlight line indicates median value for each survey. The inset is a histogram of the change in values (pre-test minus the post-test value) with the highest bar at 0, indicating no change in response.
(TIF)

S3 Fig Participant demographics and belonging. We analyzed the impact of demographic categories on changes in STEMM belonging. Outside of Previous Research (Fig 2), no other demographic categories were associated with changes in STEMM belonging. A. Underrepresented in STEMM B. First Generation College C. Socioeconomically Disadvantaged D. Year In Training E. Participation in Pre-professional Group F. Previous Research Time G. Participating in Other Summer Activity H. Seminar Participation Quantiles.
(TIF)

S4 Fig Participant post-graduate understanding. We examined student’s responses to the prompts regarding their understanding of post-graduate programs (See S2 Table for specific questions). Responses are shown based on a Likert scale, with one indicating that they strongly agreed with the statement to five indicating that they strongly disagreed with the statement. Purple highlight line indicates median value for each survey. The inset is a histogram of the change in values (pre-test minus the post-test value) with the highest bar at 0, indicating no change in response.
(TIF)

S1 Table (XLSX)

S2 Table (XLSX)

S3 Table (XLSX)
