# Supplementary figures and images for: Virtual Vanderbilt Summer Science Academy highlighted the opportunity to impact early STEMM students career knowledge through narrative
Source: PLoS One. 2021 Nov 10;16(11):e0258660. doi: 10.1371/journal.pone.0258660 (PMC8580243; doi:10.1371/journal.pone.0258660)

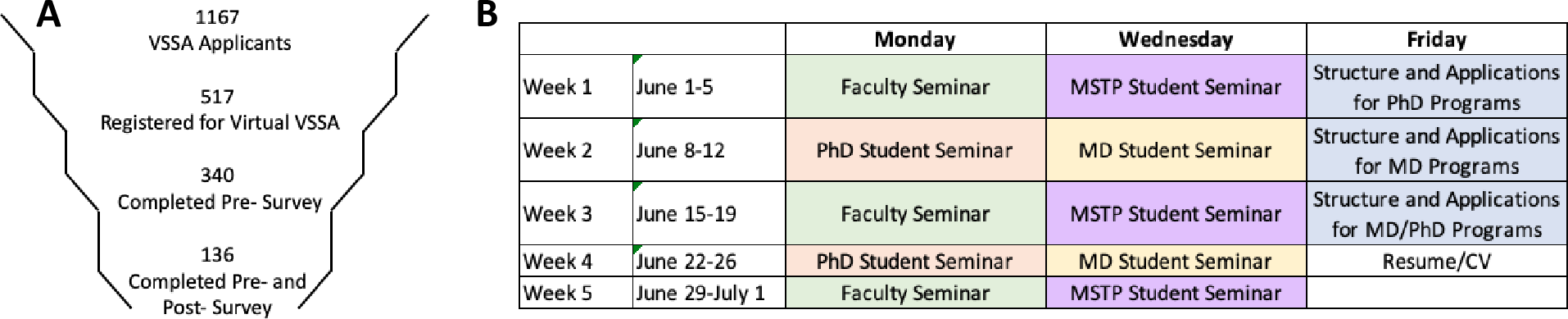

Supplement: S1 Fig — (A) To begin advertising the virtual VSSA program, the 1167 VSSA applicants were invited to participate. Of this group, 517 registered for the virtual VSSA sessions. Three hundred forty students completed the pre-survey while 136 completed both the pre-and the post-survey. This was the final population analyzed in this study, which represented just over 10% of the entire pool of VSSA students initially contacted. (B) Here we show a list of program elements by week. The program was a five-week program that began June 1st. There were five components: Faculty seminars, MSTP student seminars, structure and applications, Ph.D. student seminars, and M.D. student seminars. There was also a final presentation on resume and CV building that was delivered asynchronously. All other meetings were held synchronously. (TIF) [file pone.0258660.s001.tif]

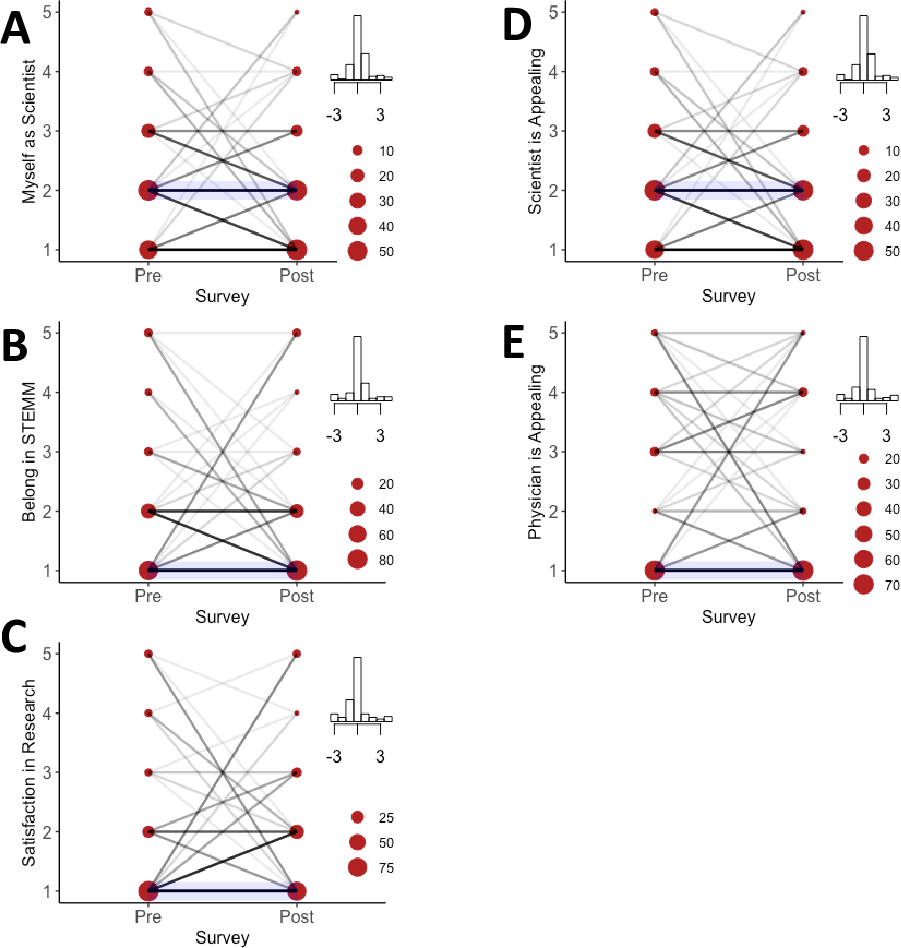

Supplement: S2 Fig — We examined student’s responses to the prompts regarding their scientific identity (See S2 Table for specific questions). Responses are shown based on a Likert scale, with 1 indicating that they strongly agreed with the statement to 5 indicating that they strongly disagreed with the statement. Purple highlight line indicates median value for each survey. The inset is a histogram of the change in values (pre-test minus the post-test value) with the highest bar at 0, indicating no change in response. (TIF) [file pone.0258660.s002.tif]

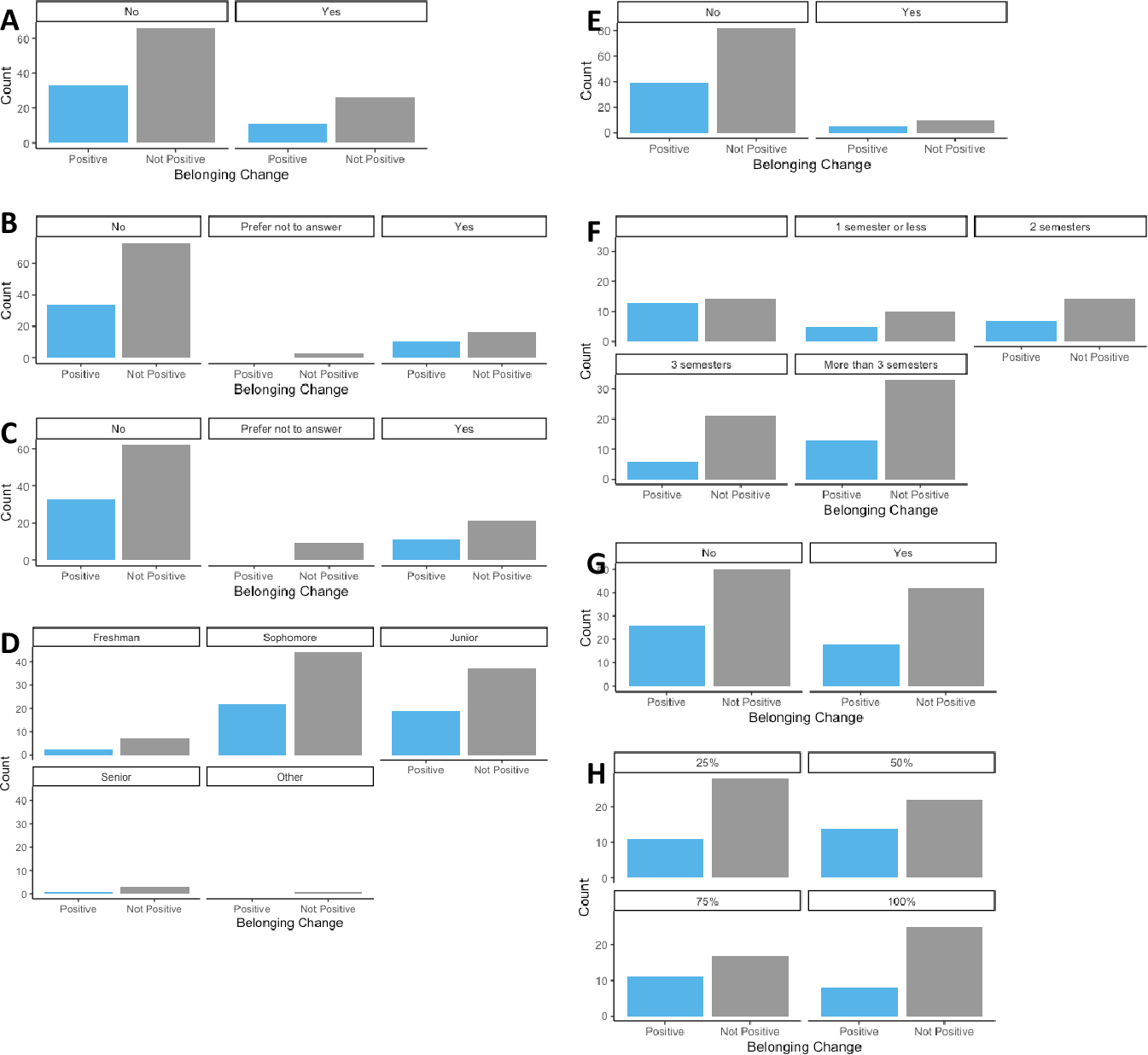

Supplement: S3 Fig — We analyzed the impact of demographic categories on changes in STEMM belonging. Outside of Previous Research (Fig 2), no other demographic categories were associated with changes in STEMM belonging. A. Underrepresented in STEMM B. First Generation College C. Socioeconomically Disadvantaged D. Year In Training E. Participation in Pre-professional Group F. Previous Research Time G. Participating in Other Summer Activity H. Seminar Participation Quantiles. (TIF) [file pone.0258660.s003.tif]

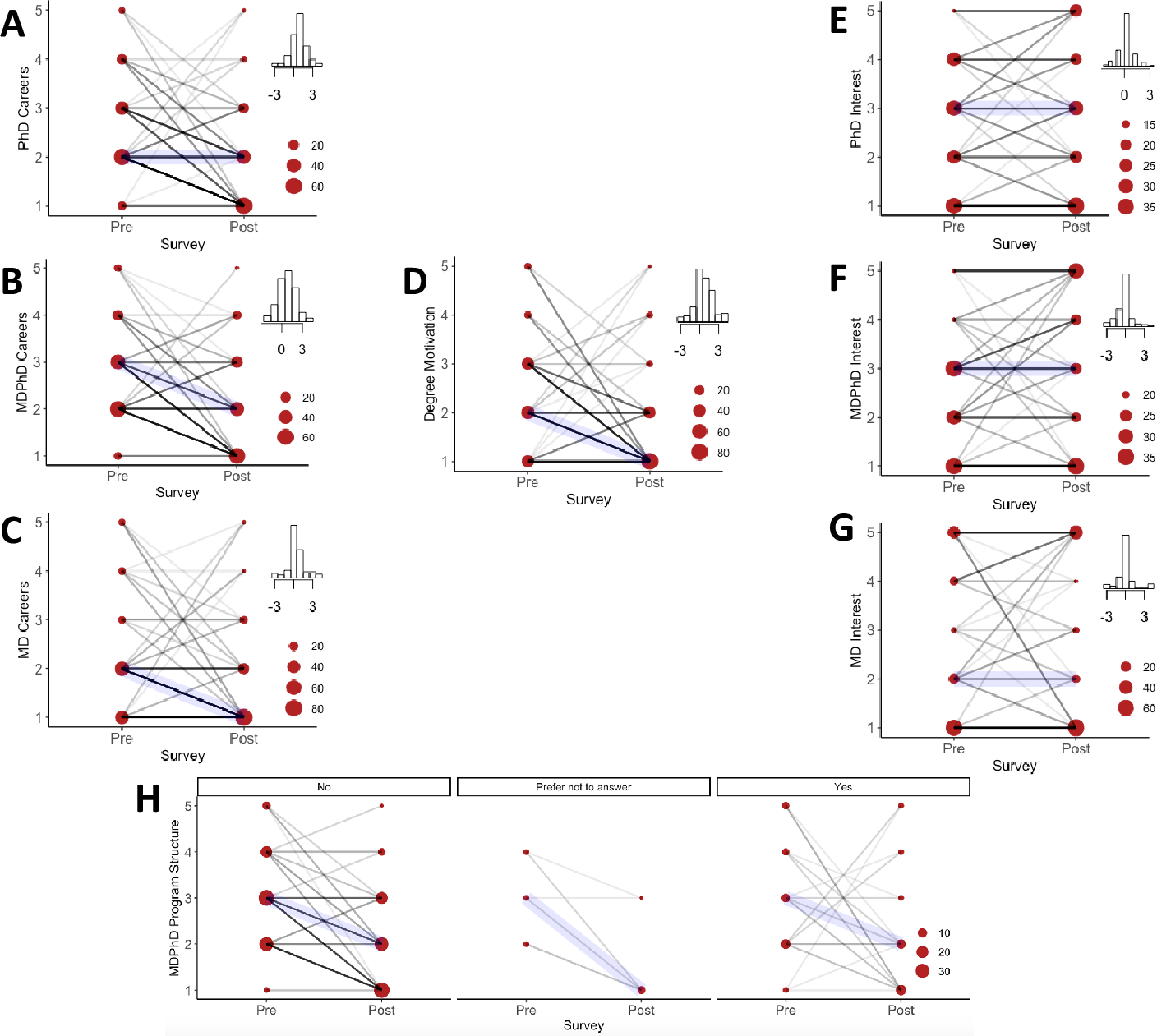

Supplement: S4 Fig — We examined student’s responses to the prompts regarding their understanding of post-graduate programs (See S2 Table for specific questions). Responses are shown based on a Likert scale, with one indicating that they strongly agreed with the statement to five indicating that they strongly disagreed with the statement. Purple highlight line indicates median value for each survey. The inset is a histogram of the change in values (pre-test minus the post-test value) with the highest bar at 0, indicating no change in response. (TIF) [file pone.0258660.s004.tif]
